# Helping Older Veterans Use Mental Health Apps: Qualitative Interviews and Development of a New Program

**DOI:** 10.2196/87361

**Published:** 2026-05-26

**Authors:** Julie Lutz, Carter H Davis, Sherry A Beaudreau, Christine E Gould

**Affiliations:** 1 Sierra Pacific Mental Illness Research, Education, and Clinical Center, VA Palo Alto Health Care System, 3801 Miranda Ave (151Y MIRECC), Palo Alto, CA, 94804, United States, 1 6504935000; 2 Department of Psychiatry and Behavioral Sciences, Stanford University School of Medicine, 401 Quarry Rd (MC 5723)Stanford, CA, 94305, United States, 1 650-498-9111; 3 Geriatric Research, Education, and Clinical Center, VA Palo Alto Health Care System, Palo Alto, CA, United States

**Keywords:** older adults, coaching, quality improvement, self-management, mobile health, smartphone, tablet

## Abstract

**Background:**

Mobile mental health apps may provide an accessible, scalable, and private avenue for older veterans who may not otherwise seek or receive care to address their mental health concerns. However, older veterans may experience barriers to using these apps that need to be addressed to facilitate effective use. Such support could be effectively implemented within the US Veterans Health Administration to facilitate the use of the United States Department of Veterans Affairs’ established mental health apps and to benefit older veterans with mental health concerns.

**Objective:**

This study aimed to (1) assess older veterans’ interest in and barriers to using mental health apps to address problems such as difficulties with social connection, and (2) develop and pilot a coaching program to address barriers that older veterans experience in using mobile devices and apps.

**Methods:**

Rapid qualitative analysis of semistructured qualitative interviews with 12 older veterans identified themes regarding interest, barriers, and preferences for support for using mobile apps. These themes informed the development of a coaching program, which was piloted with 13 older veterans to assess acceptability, feasibility, and resultant signals of changes in mobile device proficiency.

**Results:**

Most veterans expressed interest in using mental health apps. One of the most common barriers was familiarity and proficiency with mobile devices and app technology. Other common barriers included usability or accessibility of the technology or app, motivation, and memory. Veterans reported interest in receiving coaching support. Though the majority of veterans expressed some preference for more individualized and in-person support, they identified both benefits and drawbacks to all potential coaching modalities (group vs one-on-one, in-person vs remote)—including issues of individualizable and guided assistance, feasibility and accessibility of the support, and group settings as potential avenues for social connection as well as potential susceptibility to challenging social dynamics and interactions. Mobile Device and App Learning (MoDAL)—a 2-session, interactive, remote educational group—was developed and piloted. Most veterans who participated found MoDAL helpful. Participants’ mobile device proficiency showed a statistically significant improvement on average pre- to post-MoDAL, although this effect was small, and the small sample size limits the strength of the conclusions.

**Conclusions:**

Older veterans do have some interest in using mobile mental health apps to address mental health–related issues. However, they experience critical barriers, including a lack of familiarity and proficiency with the technology. MoDAL may improve older veterans’ comfort and proficiency with mobile devices and apps, which could address one of the barriers that impacts downstream engagement in mental health apps and other virtual care modalities.

## Introduction

While older US military veterans generally demonstrate resilience in mental health, a significant subset of older veterans have significant mental health concerns, such as posttraumatic stress disorder (PTSD), depression, and suicidal ideation and behavior [1]. These older veterans are less likely to engage with mental health care compared with younger veterans, but can benefit just as much when they do receive care [1]. Some of the barriers to using mental health care that older veterans experience include stigma and negative beliefs about mental health care [2]. Older adults, including veterans or nonveterans, can also present with subdiagnostic mental health symptoms, which may not be identified as needing traditional mental health care despite leading to functional impairment and/or distress [3]. Many older veterans live in rural areas, where access to care, particularly in-person care, can be challenging for those with limited resources and/or mobility [4]. Self-guided mental health management interventions have the potential to provide easy-to-access initial mental health interventions that may have lower mental health care stigma to the same extent as seeking more traditional professional care.

A potential solution to this problem may be mobile mental health apps. Mobile apps allow for an individualized, modular approach to self-management or supplemental management of various mental health and quality-of-life problems, including regulating emotions, managing distress, problem-solving, engaging in activities, social skills, etc. Given the modular nature of these apps, they could be particularly useful for highly individualized, multidimensional problems (eg, barriers to improving social connection) due to the ability to select targeted components from a varied menu of skills as appropriate based on individual needs. The United States Department of Veterans Affairs (VA) has invested in developing easy-to-access mental health apps addressing multiple targets—including PTSD, sleep, mindfulness, relationships, and everyday coping—and using components from evidence-based treatment modalities [5-7]. The apps are free to use for the general public (including veterans and nonveterans), do not collect private data, and are robustly designed and updated to enhance usability [7]. If these apps can be used to address a range of older veterans’ mental health needs, supporting engagement with these tools may have a large impact on outcomes in this population.

Meta-analyses and systematic reviews to date indicate modest but significant effects of digital health interventions, such as mental health apps, on depressive and anxiety symptoms in younger adults and in general community-based populations [8-10], especially when they incorporate cognitive behavioral therapy and mindfulness-based skills, as the VA mental health apps do. There is little reliable data on population-level age-specific differences in frequency of usage of mobile mental health apps, as well as what barriers and benefits older veterans or nonveterans experience; this is a limitation in the scientific literature, but the data to date do suggest that older adults face unique barriers to using mobile mental health apps, and may require greater attention to feasibility of using the technology and consideration of digital competence or proficiency in regard to use of digital health technologies [11]. A small sample of older adults (primarily female) identified barriers for uptake of mobile mental health interventions that included low awareness of available interventions or tools, low skill or self-efficacy with technology, likelihood of discontinuing use, preference for personal contact, and privacy concerns [12]. Older veterans experience some similar barriers to using technologies, such as mental health apps [13], in addition to potential veteran or military-specific barriers, such as hesitancy in seeking support for mental health issues [14]. The design and sustainment of VA’s mental health apps is centered on a model that takes into account quality, user experience and needs, and ongoing optimization; thus, theoretically addressing barriers on the app side such as privacy protection and usability [7]. Therefore, it appears that if support can be provided directly to older veterans to address their barriers and thereby optimize digital literacy, awareness, and older veteran preferences, older veterans may be more likely to use and benefit from these tools. Our team and others have begun this work by developing guides for using mobile devices and apps, which have been successfully used by medical providers and other health care staff to support the use of mental health apps [15-17]. This project builds upon this work to gather more detailed information on older veterans’ own identified barriers, including those that existing supports may not yet fully address.

Therefore, the first purpose of this 2-stage project was to assess veterans’ interest in and needed support for using mental health apps (part 1) as part of a project aiming to increase social connection among older veterans and explore the acceptability of and barriers to using mental health apps for this purpose. Second, we used the interview data, in addition to clinical and programmatic needs, to design a brief intervention to address one of the most commonly-identified barriers in part 1 and support older veterans’ improved familiarity and proficiency with mental health apps. We pilot tested the intervention with older veterans (part 2). This work was conducted as part of a VA Geriatric Research, Education, and Clinical Center Clinical Innovation quality improvement project that had an overarching goal of improving social connection in older veterans; thus, initial interviews discussed the use of mental health apps in the context of improving social connections, although many of the identified themes were relevant to the use of mental health apps for any primary concern. Themes specifically related to social connection itself, separate from mobile devices and apps, are presented in another paper summarizing older veterans’ needs and barriers to social connection [18]. Due to the quality improvement nature of the project, the methods and format of findings were initially designed to directly inform a specific program and setting, rather than for generalizable knowledge. However, many findings identified through this process could potentially inform additional exploration.

## Part 1: Qualitative Interviews on Interest in and Support for Using Mental Health Apps

### Methods

#### Participants

Participants for the quality improvement project were recruited from geriatrics and mental health clinics at the local VA medical center, as well as veterans who were screened as ineligible for a separate clinical trial of a stress reduction intervention at the local VA medical center. Eligibility criteria for this study were as follows: (1) aged 60 years or older, (2) enrolled in the local VA health care system, (3) adequate cognitive functioning as indicated by a total score of 9 or less on the Blessed Orientation Memory and Concentration cognitive screen [19,20], and (4) significant level of loneliness as indicated by a score of 6 or higher on the 3-item University of California, Los Angeles, Loneliness scale (UCLA), a valid and reliable, widely-used brief measure of loneliness where scores of 6 or higher are considered indicative of clinically significant loneliness [21]. Mobile device ownership was not a requirement. All questionnaires and interviews were conducted via telephone and did not require any internet-enabled device.

#### Ethical Considerations

This project was determined to be a quality improvement study and thus not research by the Stanford University Institutional Review Board (protocol 68514), and thus, documented informed research consent was not required. Referred veterans were informed as to the nature of the quality improvement project and that their participation was voluntary. Clinical Health Insurance Portability and Accountability Act (HIPAA) privacy and confidentiality protections were implemented. While audio recordings are identifiable, transcripts were deidentified.

#### Procedures

Eligible veterans were scheduled for a telephone interview. The first author, a clinical psychologist and investigator with qualitative training, completed the interview and questionnaire over the telephone. Interviews were audio-recorded with the veterans’ consent. Recorded interviews were transcribed and verified by a second reviewer for accuracy. Recruitment and interviews were stopped once the team determined that responses converged around preliminarily identified themes, with little additional information provided by each subsequent interview (ie, saturation) [22].

#### Measures

Demographics—including age, race, ethnicity, years of military service, military branch, and era of service—were collected via self-report. An interview guide was developed by the first author with input from coauthors (SAB and CEG). The guide allowed for flexibility to further prompt and explore veterans’ responses to the interview questions. The interviewer first oriented the veteran to the purpose of the interview. The first part of the interview discussed social connection; these results, along with the full qualitative interview guide, are presented in the first publication resulting from the project [18]. In the second part of the interview, the interviewer transitioned to veterans’ perspectives regarding using mental health apps. After introducing the concept of mental health apps and their potential applications for social connection, the interviewer asked open-ended questions regarding veterans’ interest in or willingness to use mental health apps for this purpose, potential barriers to using them, and thoughts about receiving coaching or support for using them and in what modalities (eg, remote vs in-person and one-on-one vs groups). The interviews were completed in approximately 30-60 minutes.

#### Analyses

Descriptive statistics were used to characterize participants. A team-based, rapid qualitative analysis approach [23-25] was used for qualitative interviews with veterans. The author team conducting the qualitative analysis was comprised of 3 independent licensed psychologists and 1 predoctoral psychology intern at the time. Furthermore, 2 separate team members transcribed and then verified each interview audio file. Then, based on the initial interview guide, a summary template was created that comprised 13 domains (6 domains pertained specifically to social connection needs and did not pertain to mobile devices or mental health apps)—the 7 domains discussed in this paper are (1) general knowledge about mobile devices and apps, (2) thoughts on using mobile apps to improve social connection, (3) barriers to using mobile apps, (4) thoughts on coaching to support using apps to improve connection, (5) potential topics for coaching, (6) modalities for coaching, and (7) likelihood or interest in participating in coaching. Next, for each interview, 1 of the team members coded all relevant quotes from interviews into summary domains, and then a second team member reviewed this organization and indicated any disagreements in how responses were coded. Consensus on appropriate categorization was reached through discussion. Finally, the first author grouped and compared responses across participants to identify subtopics and themes within each domain, and at least 1 of the other authors reviewed each domain summary. Disagreements on this cross-analysis were resolved through consensus to produce final domain and topic summaries. Although numbers of participants mentioning certain themes are presented in the results, these should not be read as dichotomous or systematic (eg, that if n=3 mentioned a theme, the remaining n=9 disagreed)—due to the open-ended nature of qualitative interviewing and analysis, some participants may not have addressed that particular theme at all, or some may have expressed multiple overlapping statements regarding the theme. Numbers are provided simply to demonstrate themes that were observed across multiple respondents.

### Results

#### Sample Characteristics

Of 47 veterans who were interested in the project and screened, 17 were eligible and scheduled interviews (n=1 scored >9 on Blessed Orientation Memory and Concentration cognitive screen, n=29 scored <6 on UCLA). Moreover, 4 did not attend or reschedule their interviews; thus, 13 interviews were conducted. One of these 13 veterans refused to consent to audio recording, resulting in poorer data quality due to the interviewer’s limitation to concurrently typed notes; therefore, this interview was not included in the thematic analysis. Per the authors’ judgment, saturation was reached by the 13th interview based on redundancy of themes; via preliminary judgment or summary of apparent major themes by the interviewer (JL) throughout the conduct of the interviews, no themes significantly divergent from previous interviews were identified within the last 2 interviews. This sample size was consistent with data demonstrating the possibility of reaching saturation within approximately 12 interviews [22]. All participants were men. The demographic and military service characteristics of the sample are presented in Table 1. Participant numbers were assigned randomly.

**Table 1.** Characteristics of veterans participating in part 1 qualitative interviews and part 2 MoDAL^a^ pilot (4 participants in part 1 also participated in part 2).

Characteristics	Part 1: Qualitative (n=12)	Part 2: MoDAL pilot (n=13)
Age (y), n (%)		
60‐69	4 (33)	4 (31)
70‐79	6 (50)	8 (62)
80 or older	2 (17)	1 (7)
Age (y), mean (SD; range)	72.3 (7.7; 60-82)	72.6 (6.4; 62-80)
Race or ethnicity, n (%)		
Part 1^b^		
White, non-Hispanic or Latino	10 (83)	—^c^
Other, Hispanic or Latino	2 (17)	—
Part 2^b^		
White	—	8 (62)
Black or African American	—	2 (15)
Native American	—	1 (8)
Chose not to respond	—	2 (15)
Time in military (y), range	2‐25	Not collected
Military branches represented	Army, Navy, Marines, Air Force, and reserves	Not collected
Military eras^d^, n (%)		
Vietnam era (approx. 1964‐1972)	8 (67)	Not collected
Other (eg, later 1970s, early 1980s, and Gulf War era)	6 (50)	—

a MoDAL: mobile device and app learning.

b Data collection processes and format of demographic data differed between parts 1 and 2.

c Not applicable.

d Some Veterans served in more than one era.

Below is a description of themes identified in the qualitative interviews. Within the 6 broad domains of the interview—knowledge, thoughts on using mobile apps, barriers, feelings about coaching, potential topics, modalities, and likelihood of using coaching—we identified several themes to inform supports provided for use of mobile mental health apps among older veterans. Although older veterans reported some level of general knowledge of mobile devices and apps, many reported limited knowledge. Most saw mental health apps as potentially helpful for supporting social connection, but several barriers to using them were identified. Finally, many veterans endorsed using coaching to overcome those barriers and gave suggestions for the best areas of focus for support.

#### General Mobile App and Device Knowledge and Experience

There was a large range in self-reported knowledge and self-efficacy in using mobile devices and apps. All reported broad or general knowledge about mobile devices and what mobile apps are, but many (n=4) stated that their knowledge of and skills with apps were quite limited (“[referring to using apps/devices] Yeah, I’m not really any good at that.” [Participant 7]). Some veterans (n=3) reported more knowledge, as well as a sense of self-efficacy in learning to use technology better over time (“I can navigate my phone pretty good.” [Participant 5]). Although some reported not being aware at all of VA mental health apps (n=2), several (n=4) stated they were aware that they were offered, although most did not have detailed knowledge about them. Moreover, 2 were familiar with at least 1 VA mental health app or with the types of skills offered in them (“I think the mindfulness one.” [Participant 7]). Furthermore, 5 veterans expressed that while their knowledge or use of their devices was relatively limited, they had familiarity with specific apps or functions that they used more frequently. Examples included mobile games, streaming services, exercise or sports accompaniment apps, and banking apps.

*I usually just use my phone for TV and YouTube or when you need an app to really interact with your finances. So this one here… it would take me time to get through it… to see what the platform is like.*

[Participant 12]

Of the total, 6 veterans spontaneously commented explicitly in the interview on owning or using at least 1 mobile device (smartphone or tablet), including devices provided through the VA telehealth program (“I had frequented a tablet I was used to, but not being filled in the reuse or use of it. It became foreign to me.” [Participant 6]); this question was not directly asked as part of the interview, and thus more may have owned or used at least 1 mobile device.

#### Using Mobile Apps to Improve Social Connection

Veteran perspectives about whether mental health apps could potentially be helpful in improving social connection were sought to ascertain their openness to using mental health apps to address broader life problems or mental health. Almost all veterans (n=11) explicitly expressed some willingness to attempt using mental health apps to help improve social connection. Some (n=4) expressed a hesitant willingness to try the apps with qualifications about potentially stopping if no benefit (“I would have to simply see how it works in that particular case… I’d be open to trying it, yes.” [Participant 1]). Whereas others (n=2) stated that using the apps would be “fun” (“Would be fun, would be something to do.” [Participant 3]). Notably, some veterans appeared to conflate social networking–type apps with the individual-focused mental health apps to improve social skills and/or address internal barriers to connection.

There was also a large range in veterans’ beliefs about how useful the apps could be in helping to improve social connection, with many (n=3) reserving their opinions about the potential helpfulness until after trying the apps.

*They may not hit the nail on the head, but they may be useful… and you don’t know, they may be somewhat useful.*

[Participant 1]

Some expanded on specific situations in which mental health apps may be more or less helpful; 2 posited that the apps may be less helpful for depression, anhedonia, or lack of motivation compared with anxiety. Relative potential benefits of apps were helping veterans directly connect with others, or providing more convenience than formal mental health care.

*I would probably not go to that as my first stop, if I was going through… an isolated period or a period of depression or a period of… non-connection. I’m not sure if that would be my first, you know my first thought.*

[Participant 8]

#### Barriers to Using Mobile Apps

Comments regarding barriers to using mobile apps frequently referred to the broad use of mobile apps, not necessarily specifically mental health apps or using apps for social connection. The most common barrier (n=8) to using mental health apps that veterans identified for themselves or others was the level of knowledge, skill, and comfort with using mobile devices and apps. Several veterans (n=4) commented on age and generational differences in familiarity with technology, and a likely learning curve for many older veterans. Moreover, 2 also expressed concerns that technology could negatively impact social behaviors.

*If you’re dealing with a person close to 80, like me, or a person 60. There’s a huge difference there… So someone in my age finds it extremely difficult to maneuver these things… versus a person that’s 60… They breezed through that like it’s just standing still. That 20 years is huge.*

[Participant 11]

In total, 6 veterans mentioned usability factors that could significantly impact veterans’ engagement with and effectiveness of the apps, including easy-to-learn functionality, accessibility for those with sensory impairments, functionality with poorer internet connections or older devices, and careful assessment and updating of apps based on user experience.

*The software coders that develop these apps aren’t necessarily the users of them. And they often times are severely lacking in real functionality… A lot of the older people are going to be having difficulty using the device because they can’t see it.*

[Participant 10]

Furthermore, 5 veterans expressed that motivation to use the app could be a significant barrier, such that tactics to engage the veterans may increase use.

*If it’s an app that says come back and do the exercise… once a week, twice a week, daily, give it 10 minutes of your time once a day… or if it’s just something that sits there and says, yeah, I’m on your phone, but I’m not going to entice you in any way to check this out, then you’re liable to just forget about it, honestly.*

[Participant 10]

Of the total, 4 veterans stated that memory can get in the way of learning and keeping track of where their device is (“Because like I tell you, they explain it to me, and then… I turn around and forget all about it.” [Participant 3]).

#### Coaching for Using Mobile Apps

When presented with the concept of having a coach to help support the use of mental health apps to address social connection, 6 veterans stated that having a coach to help learn and navigate the apps could be helpful (“[referring to coaching] Yeah, that would be good. Just like having a mentor or something to kind of touch bases with you.” [Participant 12]). Moreover, 4 veterans identified strategies that a coach could use for improving the use of mobile apps, including providing written notes to help with memory, demonstrations of how to use the device or app, helping the veteran focus, and using repetition to support memory.

*Someone showing me and… helping me work out how to use it [mobile app] the best for me.*

[Participant 7]

Furthermore, 5 veterans highlighted skills the coach would need to be effective, including avoiding ageism (eg, no elder-speak: “[No] talking to them as if they’re a child.” [Participant 10]), positive social skills, ensuring the training or coaching is interactive and engaging, and using an appropriate level of vocabulary and speed.

*You can’t have a friend explain that to you that is really good with it because… they’re talking so quickly… about the technical words that I have never even heard before…It would be like a 747 going over somebody’s head.*

[Participant 11]

#### Topics for Coaching

Veterans identified several topics that they would want to be covered in coaching for using mental health apps. General proficiency with using mobile devices and apps was identified by four veterans as the first necessary step (“Yeah, basic and go from there.” [Participant 11]).

In total, 5 veterans expressed a desire to learn more from their coach about specific skills that could be addressed by the apps, such as mindfulness, coping with triggers, adjusting to changes since COVID, and social skills (“An app that would help me divert triggers.” [Participant 2]). In addition, 2 veterans mentioned a desire to learn more about social connection more broadly (“[talk about] my impediment to develop social interactions.” [Participant 9]). Finally, 2 veterans discussed their desire to be provided with information about or be pointed to which mental health apps are available.

*Say, “Here’s where we’re going with the program. And this is what we got to offer you. And we’re gonna start here and work our way towards that goal.*”

[Participant 4]

#### Coaching Modality

Veterans were asked about their opinions and preferences regarding remote phone or video coaching versus in-person and individual one-on-one coaching versus in groups. One veteran (Participant 1) did not directly respond to these questions regarding modality, and another (Participant 5) responded regarding remote versus in-person but not individual versus group modalities. Responses were diverse, with most veterans indicating openness to various or multiple modalities. Regarding remote or in-person, more than half (n=6, 55% of respondents) were open to both remote and in-person; 9 out of 11 (82%) did express some kind of preference regardless of openness to other modalities, where 6 (67% of those with a preference, 55% of respondents) preferred in person, 2 (22% of those with a preference, 18% of respondents) preferred video, and 1 (11% of those with a preference, 9% of respondents) preferred phone. Regarding individual or group, 80% of respondents (n=8) were open to both individual and group; 7 out of 10 (70%) did express a preference regardless of openness to other modalities, where 6 (86% of those with a preference, 60% of respondents) preferred individual and 1 (14% of those with a preference, 10% of respondents) preferred group. Among those expressing preferences regarding modality, many preferences were relative to the other options, with participants indicating that they were open and willing to participate in other modalities. Most interviewees identified the benefits and drawbacks of multiple or all modalities.

Regarding remotely delivered coaching, the main “pro” of video or phone delivery was accessibility. The “cons” included the difficulty of teaching technology remotely when participants are already not familiar with it (“You can’t use the video or the phone to teach somebody something if they don’t know how to use it.” [Participant 10]). Phone was generally believed to be easier, but video was seen to be more conducive to interaction and connection (“I am getting more comfortable with the video chat because it seems like you have contact with somebody. And that seems to be the need is to have contact with somebody.” [Participant 4]). Veterans stated video delivery would require proper orientation to the video interface first, and must be easy to join. Veterans stated that in-person delivery was best for connection and learning, and could help them “get out” more, but that it is less accessible and requires more careful timing to allow veterans to attend (“I think that it would be more difficult to develop excuses in person rather than on the video or some other means.” [Participant 9]).

Regarding individual versus group coaching, the pros of one-on-one coaching were potentially a deeper connection with the coach, easier concentration and fewer distractions, and less concern about embarrassment or stigma. A con was no connection with other veterans. Veterans stated that groups could allow them to help each other. However, they commented on potential barriers such as personality, knowledge, and experience differences; others dominating a group; and self-consciousness. Veterans recommended that groups be small (eg, 8 or fewer) and that leaders be skilled at moderating and keeping the group on track.

*There’s nothing worse than a participant on that zoom call for an hour… that never gets a chance to speak his or her mind, and a lot of these zooms are taken over… by the most boisterous or… the person that they feel are you know, that they’re the most severe case.*

[Participant 8]

Veterans’ preferences for frequency ranged from every day to begin with, to weekly or biweekly, to once a month (“And I think personally for me, once a week thing at a designated time with a designated agenda would work best for somebody like myself.” [Participant 8]). Veterans did not narrow down a specific number of sessions or total time in coaching, mainly stating that it would depend on how long it takes an individual to learn the information.

#### Likelihood or Interest in Participating in Coaching

In total, 10 veterans expressed interest in trying a coaching intervention. Of these, 4 used stronger language such as “certainly,” “more than willing,” and “very likely.” Most stated that ongoing participation would depend on perceived helpfulness. Furthermore, 2 veterans did not provide responses directly regarding interest.

*I’ll go at least once. And then I’ll see if it seems to be working. And then… I’ll probably make myself go more if I think it’s working.*

[Participant 7]

#### Translating Qualitative Results to Intervention Development

**Figure 1.**
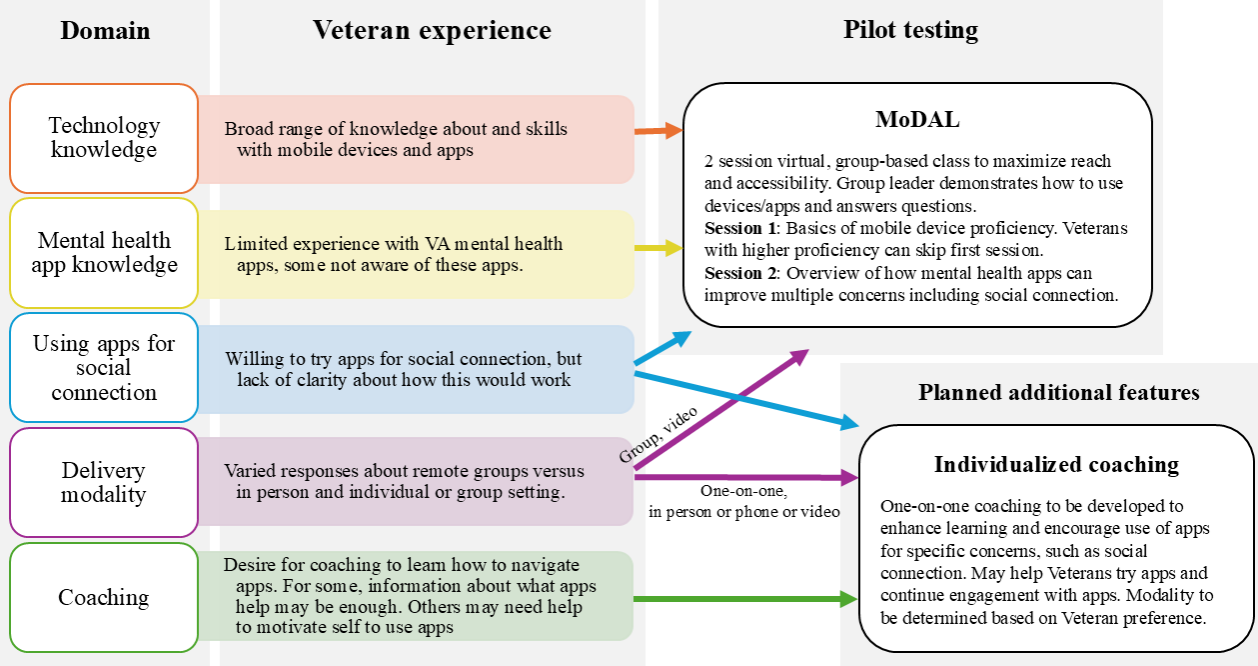
Summary of findings from qualitative interviews and translation to MoDAL development and piloting and planned additional intervention features. MoDAL: Mobile Device and App Learning; VA: United States Department of Veterans Affairs.

The above findings were incorporated into developing a stepped care program to address barriers to older veterans’ use of mental health apps to address social connection and other concerns. A stepped care model [26] allows for different “levels” of mental health intervention based on the level and type of need for the veteran. Veterans with more basic foundational support needs, such as learning to use devices and apps, may participate in an education intervention. This basic foundation may be all the support needed for some veterans, who may then go on to independently use the technology. Others may need more specific and guided additional support. Given veterans’ openness to diverse modalities for support, nuanced discussion of benefits and drawbacks to each modality—including recognition of accessibility and feasibility factors—and varying levels of support needed, the stepped program includes a mix of modalities and offers various levels of support. An initial brief educational intervention is delivered remotely via video to groups to maximize accessibility and reach, with a planned future one-on-one coaching step that can additionally explore individual veterans’ unique needs and barriers in their preferred modality (Figure 1).

The first step of the program—Mobile Device and App Learning (MoDAL)—was developed to address key barriers identified by veterans in part 1. From the multiple barriers identified by veterans in part 1, we identified proficiency and self-efficacy with mobile devices and apps as the most foundational barriers to be addressed in the intervention. The selection of this barrier to address is supported by work on the Unified Theory of Acceptance and Use of Technology and eHealth literacy across the lifespan, which demonstrates that addressing health technology literacy is critically linked to discrepancies in eHealth use in older adults [27]. Other concerns, such as memory, were partially addressed using handouts provided to participants to support their learning about mobile devices. Usability concerns (eg, accessibility for sensory impairments) were incorporated into the intervention through sharing information about settings that could improve usability (eg, adjusting font sizes).

MoDAL was piloted in part 2 of this quality improvement project. We plan to add future, more individualizable intervention steps to then address the additional barriers, such as motivation, that may prevent older veterans from engaging with mobile mental health apps. Although MoDAL was developed with a long-term aspirational goal of increasing older veterans’ use of mental health apps, the immediate goal of MoDAL is to address the specific barrier of proficiency and familiarity with mobile devices and apps; this program is not purported to be sufficient alone to increase app use.

## Part 2: Piloting MoDAL

### Methods

#### Overview

We developed and iteratively piloted a brief intervention to teach older veterans how to use mobile devices and apps, called MoDAL. Although veterans generally somewhat preferred in-person and one-on-one modalities for coaching in part 1, most recognized the potential benefits of and were very willing to try remote and group modalities for feasibility and for potential connection with other veterans. Aligning this with institutional or practical needs for scalability and reach for this initial support step, MoDAL was designed to be a 2-session (90 min each), interactive group video class. Refer to Figure 1 for a summary of how qualitative interviews informed MoDAL development and how MoDAL was conceptualized as a part of a stepped-care approach. The second step, to be developed and piloted next, will address and incorporate the veterans’ identified benefits of one-on-one support in a feasible way targeted to those in need of a greater level of support.

The first session of MoDAL provided basic information about using mobile devices (ie, smartphones and tablets) and mobile apps broadly—main objectives or topics included identifying universal symbols commonly used on devices or apps, reviewing critical and commonly-used functions, learning how to adjust settings for improved usability (including accessibility settings for various sensory and physical limitations), and understanding best safety practices (eg, securing devices and information security online). The second session reviewed the mental health apps available from VA and how they could be used to self-manage various problems including social connection, and it included a detailed interactive demonstration of the most-used app—PTSD Coach (Department of Veterans Affairs)—which has been used in VA clinical trials and has a large body of research support [28]—other than the demonstration, main objectives or topics included reviewing the general purposes of all major VA mental health apps, identifying how different skill modules in apps may apply to different types of problems, and understanding the security of VA apps. Some of the questions around the use cases for mental health apps identified in the qualitative interviews (eg, confusion about mental health apps that can address barriers to social connection vs social networking apps) were incorporated into the content. Both classes involved visual slides, interactive activities (eg, matching game regarding meanings of common device symbols), screen-shared demonstrations on the instructor’s own device, and walkthroughs to try on their own mobile device. To allow veterans to follow the instructor while trialing the app on their own device, veterans were also encouraged to join the class on a computer or other device if possible, so they could follow along on their mobile device during the class. All participants were also mailed packets with many resources and references, including printouts of the MoDAL slides; brochures for several VA mental health apps; handouts describing how to log in and use the VA Video Connect platform through which classes were held; and step-by-step guides for using the 2 major types of device platforms, how to download apps, and using PTSD Coach. Materials and content were adjusted between groups based on instructor observations and veteran feedback. For example, adjustments included changes to slide layout and content, in-group activities, and print materials provided in the mailed packet. In these groups, the instructor was the first author (JL), who was also the primary developer of the program and is a doctoral-level clinical psychologist; the instructor or developer reviewed several existing resources (including those created by last author [CEG] [15]) highlighting critical use cases and functions of major device operating systems and VA mobile apps, and created a preliminary working draft of a training manual for future instructors. The 2 MoDAL sessions were delivered with a 1-week interval. To request MoDAL materials or further information, please contact the authors.

#### Ethical Considerations

This project was determined by the Stanford University Institutional Review Board (protocol 68514) and VA Palo Alto local Research & Development to be quality improvement and thus not research; hence informed consent was not required. Referred veterans were informed as to the nature of the educational program, and participation was voluntary. Clinical HIPAA privacy and confidentiality protections were implemented.

#### Procedures

Veterans who participated in the qualitative interviews were invited to participate in MoDAL groups. Groups were also advertised via flyers and tables at events at the local VA, and referrals were sought from outpatient mental health and primary care providers, as well as the same research clinical trial on a stress reduction intervention as in part 1. Eligibility to participate in MoDAL was opened as much as possible with the long-term goal of accessibility, inclusivity, and utility for as many veterans as possible. Veterans were eligible to participate in MoDAL if they were aged 60 years or older, enrolled in the local VA health care system, and could appropriately participate in the group (eg, no disruptive behavior and adequate cognitive functioning to participate and benefit). They were also implicitly required to own or have access to a mobile device or computer to join the group. Because the primary focus of MoDAL was on learning about devices and mental health apps, there was no inclusion or exclusion criterion regarding the level of social connection. Additionally, veterans were allowed to participate in only 1 of the 2 MoDAL sessions if desired, as a purposeful intervention design choice to handle real-world, self-identified needs and variation in baseline knowledge. For example, if a veteran felt they were already knowledgeable about general device usage, but wanted to learn more about the VA apps, they could participate in just the second session. Groups were scheduled at intervals when at least 2 veterans and up to 8 veterans could be gathered. Before the first session, veterans completed questionnaires over the phone administered by a clinic scheduler. The first author led the MoDAL groups. Following the second session, the veterans again completed questionnaires—plus feedback about the program—over the phone with the scheduler.

#### Measures

Participants were asked about their age, gender, and race or ethnicity in a free-response manner before MoDAL. They were also asked what type of mobile device platform or operating system they had. Though the primary focus of MoDAL was not specifically social connection, we did administer the 3-item UCLA [21] (each item scored 1 to 3, totaled for a score ranging from 3 to 9, with higher scores indicating greater loneliness) before and after MoDAL to assess if participation in the MoDAL group was associated with any change in connection.

The Mobile Device Proficiency Questionnaire—16 items (MDPQ-16) was administered before and after MoDAL to assess familiarity and proficiency with smartphones and tablets; it has demonstrated good reliability and validity [29]. The 16 items are comprised of 8 sets of 2 items each, measuring the respondent’s self-reported ease with using various functions of the device (eg, navigating menus and keyboard, sending emails, finding information, and using media). Each item is rated 1 to 5 regarding its ability to perform each task (1=never tried, 2=not at all, 3=not very easily, 4=somewhat easily, and 5=very easily), and scores on the 2 items within each set are averaged, and those averages across the 8 sets are totaled.

After MoDAL, veterans were asked for feedback on the program via 2 rating items and 2 free-response items. Rated items assessed helpfulness of the program for becoming more familiar with mobile devices and apps, and helpfulness of the program in learning more about VA mental health apps. Ratings were from 1 to 4 (1=“very unhelpful,” 2=“unhelpful,” 3=“helpful,” and 4=“very helpful”). Free response items were (1) “What did you like most about the program?” and (2) “What would you change about the program to make it better?” The scheduler conducting the assessment wrote notes on free responses—responses were not audio-recorded nor perfectly transcribed.

#### Analyses

Descriptive (eg, frequency and mean) analyses were conducted on demographics to characterize the sample. Frequencies of participation and completion of one or both sessions were also recorded. Repeated measures *t* tests were conducted to examine the change from pre-MoDAL to post-MoDAL on the MDPQ-16 and UCLA. Frequencies of ratings on post-MoDAL program ratings are provided, as well as a brief informal summary of responses to the 2 open-ended questions.

### Results

In total, 13 veterans participated in at least 1 MoDAL group session. Participants were all men. Of the total, 4 (31%) participants were veterans who had participated in the qualitative interviews. Refer to Table 1 for a summary of other sample demographics. All veterans owned or had access to a smartphone and/or tablet running one of the top 2 operating systems. Moreover, 4 groups were held between April and October 2024, ranging in size from 2 to 4 veterans. Of the total, 10 veterans completed both MoDAL sessions, 1 completed the first session only, and 2 completed the second session only.

**Figure 2.**
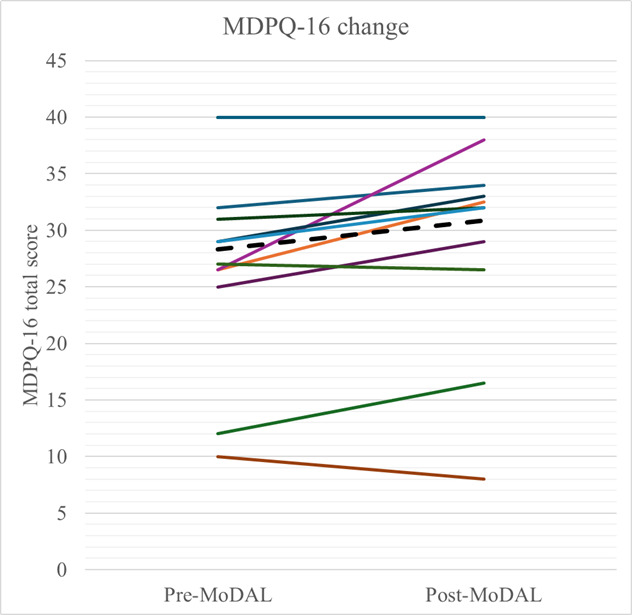
Change in MDPQ-16 scores pre- to post-MoDAL for 13 participants. Dashed black line is sample mean. MDPQ-16: Mobile Device Proficiency Questionnaire–16 items; MoDAL: Mobile Device and App Learning.

Mobile device proficiency scores on average exhibited statistically significant change from pre-MoDAL to post-MoDAL (2-tailed *t*_12_=2.71; *P*=.02; pre: mean 28.31, SD 9.36; post: mean 30.89, SD 9.45). Refer to individual trajectories in Figure 2. Cohen *d* effect size of this difference was large, but with a large confidence interval due to low power or small sample size (Cohen *d*_RepeatedMeasures_=0.72, 95% CI 0.10-1.33); we caution against overinterpretation or generalization of this effect size given the very small sample size and no comparison group or randomization. Additionally, there was heterogeneity in response (n=3, 23% veterans demonstrated minimal or small improvement, n=5, 38% exhibited larger improvements [≥4 points, or about a 10% increase], n=3, 23% exhibited no improvement [these represent a ceiling effect as all had baseline scores of the maximum 40], and n=2, 15% exhibited a small decline [up to 2 points]). There may also be some heterogeneity due to partial compared with full participation; although we do not provide grouped means by partial versus full participation due to the very small sample size of partial completers (n=3), the ranges of change in MDPQ-16 for partial completers were from −0.5 to 4, and for full completers were from −2 to 11.5. Loneliness scores did not exhibit significant change (*t*_12_=−0.58; *P*=.57; pre: mean 5.00, SD 1.87; post: mean 4.77, SD 1.69).

As illustrated in Table 2, most veterans rated the program as either helpful or very helpful regarding using mobile devices and apps (11/13, 85%) and regarding learning about VA mental health apps (12/13, 92%). In free response items, veterans identified various aspects they liked most, including the hands-on step-by-step approach, learning new things, becoming more familiar with VA apps, and the instructor being interactive and helpful. Regarding potential improvements, 6 (46%) stated they had no suggestions for improvement; others suggested adjusting the program based on the premeasured knowledge level of the group, making more accommodations for those with sensory impairments (eg, adjusting activities for those with visual impairment), and improving the support provided for issues with using the secure videoconferencing platform.

**Table 2.** Satisfaction ratings (total n=13) of veterans who participated in MoDAL^a^.

Helpfulness of program in…	Rating, n (%)			
	Very unhelpful	Unhelpful	Helpful	Very helpful
Becoming more familiar with mobile devices and apps	0 (0)	2 (15)	7 (54)	4 (31)
Learning more about VA mental health apps	0 (0)	1 (8)	5 (38)	7 (54)

a MoDAL: mobile device and app learning.

## Discussion

### Principal Findings

Overall, older male veterans were open to trying to use mental health apps to improve social connection and other mental health–related problems. Skepticism about whether veterans would use the apps consistently or find them helpful was common, and veterans’ responses echoed what was found in a previous study of barriers to using digital mental health interventions among older adults, which found that participants were likely to discontinue use if the technology was too difficult to use or outcomes were not satisfying [12]. Another consistent finding was the concern about self-efficacy with using the technology as a significant barrier to uptake of this type of intervention. Veterans generally agreed that coaching support for learning how to use mobile device and app technology could facilitate mental health app use. They were generally receptive to trying coaching remotely or in person, and in one-on-one or group settings, with appropriate considerations made (eg, making content engaging, managing the group to prevent monopolization by any 1 veteran, and making activities and content accessible for those with sensory or functional impairments). Responses showed older veterans did consider access and stigma related to receiving support for social connection or mental health—previously identified as potential barriers to care [2]—and identified that mobile apps could increase accessibility and be acceptable in the face of stigma. Between past research demonstrating potential positive effects of digital mental health interventions [8-10] and older veterans’ own perception of possible effectiveness, this is an avenue worth further attention in older veterans’ mental health care.

Based on benefits and drawbacks identified by veterans including accessibility and difficulty traveling, and potential social benefits of a group, in addition to practical considerations of feasibility and reach, we developed a brief interactive group coaching class delivered by video over 2 sessions, to teach older veterans how to use mobile devices and apps and familiarize them with the VA’s mental health apps, as the first step in a stepped-care program that would incorporate diverse modalities to meet diverse needs. Veterans who participated in these groups generally provided positive feedback regarding acceptability and perceived helpfulness. Additionally, even in this small pilot and in a sample that started with moderate levels of device proficiency at baseline (about 28/40), veterans reported some improved mobile device proficiency, although the size of this improvement appeared to be rather small—unfortunately, there are no clear data on clinically meaningful scores or changes on the MDPQ-16.

This quality improvement project and the findings reported here will inform further development, efficacy testing, and implementation of MoDAL, such as through a stepped-care approach providing a continuum of minimally intensive and group or self-guided to more intensive and one-on-one levels of intervention as needed per individual [28] for mental health and behavioral problems, such as social connection, anxiety, and mood problems. These data demonstrate preliminary feasibility, acceptability, and a signal for potential improvement in mobile device proficiency with MoDAL. The next step is establishing efficacy via rigorous, fully-powered efficacy research, including translation to app usage—the longer-term, aspirational goal of addressing these barriers is to increase older veterans’ engagement with mental health apps. Thus, as part of establishing efficacy and broader utility and during implementation, examination of frequency and sustainment of app use after participation in MoDAL and other stepped interventions is necessary. Previous work on the Unified Theory of Acceptance and Use of Technology and eHealth literacy [27] supports the hypothesis that MoDAL’s impact on proficiency may indeed play a role in increased uptake of mental health apps among older veterans. However, if efficacy is confirmed, there are several areas for potential growth in the program. As highlighted in Figure 1, an added option for more individualized brief one-on-one coaching following MoDAL, specifically focused on using mental health apps to improve the veteran’s primary concern, could benefit those with greater support needs. This approach would optimize the benefits of different modalities and address individual support needs that veterans identified here while remaining scalable within VA. Individualized or one-on-one coaching has been demonstrated to be feasible and satisfactory to older veterans for improving mobile device proficiency and app use [17], and can be used as necessary for those with greater support needs or complex cases. MoDAL could also be scaled up in the future by employing peers as group leaders or coaches and expanding referral sources to include VA programs such as digital divide (which helps rural and older veterans access internet and devices) and whole health (which emphasizes veterans’ values and a well-rounded view of health across biopsychosocial domains).

Of course, this small pilot quality improvement project has limitations. This MoDAL quality improvement program was a single-arm pilot of feasibility, not randomized or controlled, preventing strong conclusions regarding direct effects. Both samples were 100% male, and therefore, conclusions cannot be made regarding the applicability of these findings to female veterans. Third, the necessary context provided during the qualitative interviews to prompt the veterans to think about the potential utility of mobile apps for addressing social connection could have introduced bias in responses; describing the potential ways apps could address social connection barriers may have led to an iatrogenic or artificial elevation in opinions about the potential benefit of apps. We made many efforts to encourage candidness and did indeed observe that our veterans were candid about potential concerns throughout the interviews. Inclusion criteria also changed somewhat from part 1 to part 2 (no loneliness requirement and no formal cognitive assessment)—which may have impacted the direct translation of themes identified in part 1 to the development and implementation of MoDAL in part 2 to some extent, such that the targeted populations may experience somewhat different barriers to using mental health apps. Additionally, MoDAL was limited to remote group-based delivery at this time for feasibility; although veterans generally demonstrated openness to both individual- and group-based modalities, as well as both in-person and remote, there was some preference for one-on-one in-person support, and some veterans may potentially find remote and/or group delivery more challenging or less preferable. Despite this, veterans who participated in MoDAL found it helpful. Furthermore, the added conceptualized stepped support as proposed in Figure 1 would allow for different types of modalities that fit the unique needs of different veterans. Also, at this time, MoDAL was only provided via video, and therefore, the pilot included only veterans who were willing and able to participate by video. Initial or baseline proficiency scores were within a moderate range for the most part, suggesting that this pilot did not necessarily capture the most mobile technology-naïve veterans. Future testing of the intervention in an even less proficient population will be necessary. Data were not collected on exact dates of post assessments; thus, there may have been some variation in the length of time from the second MoDAL session to post-MoDAL ratings of proficiency and satisfaction, which could potentially impact recall. Participants in subsequent MoDAL groups may have experienced slightly heterogeneous interventions, due to the iterative nature of the design; however, changes between groups were quite small, usually on the level of adding or removing 1 or 2 functions that veterans in a previous group stated were helpful or not helpful to review. Additionally, there may be heterogeneity in intervention dose or response due to the design allowing for partial participation in any 1 or both sessions. At this time, MoDAL required device ownership for participation as the project did not have funds to provide devices. For greater impact in the future, the provision of connected devices would be greatly beneficial to increase reach and accessibility—the VA digital divide program currently provides one avenue through which veterans without devices or internet connection can receive necessary support [30]. Finally, due to the limited scope of this project as a quality improvement project focused on initial development and feasibility, these data could not be used to formally examine issues such as heterogeneity in response to MoDAL, impact of full versus partial participation, objectively measured mobile device or app proficiency rather than self-report, etc.

### Conclusion

More development and controlled research will be necessary to establish the efficacy of this program in a larger sample of older veterans. Implementation research on barriers and facilitators for intervention delivery and scalability will also be needed. Such implementation research may benefit from using an evidence-based, structured framework, such as the Non-Adoption, Abandonment, Scale-Up, Spread, and Sustainability framework, to guide and examine complexity in barriers and facilitators to implementation [31]. This framework, in particular, has been effectively used in digital intervention work [32]. Overall, feedback from older veterans as well as the feasibility and acceptability of the MoDAL program highlight the need and potential utility for innovative and accessible means of promoting mental health app proficiency and efficacy in this population.

## Supplementary material

10.2196/87361 Checklist 1 SQUIRE checklist.

## Abbreviations

HIPAA: Health Insurance Portability and Accountability Act
MDPQ-16: Mobile Device Proficiency Questionnaire–16 items
MoDAL: Mobile Device and App Learning
PTSD: posttraumatic stress disorder
UCLA: University of California, Los Angeles, Loneliness Scale
VA: United States Department of Veterans Affairs
